# Assessment of Second-Opinion Strategies for Diagnoses of Cutaneous Melanocytic Lesions

**DOI:** 10.1001/jamanetworkopen.2019.12597

**Published:** 2019-10-11

**Authors:** Michael W. Piepkorn, Gary M. Longton, Lisa M. Reisch, David E. Elder, Margaret S. Pepe, Kathleen F. Kerr, Anna N. A. Tosteson, Heidi D. Nelson, Stevan Knezevich, Andrea Radick, Hannah Shucard, Tracy Onega, Patricia A. Carney, Joann G. Elmore, Raymond L. Barnhill

**Affiliations:** 1 University of Washington School of Medicine, Seattle; 2 Program in Biostatistics and Biomathematics, Fred Hutchinson Cancer Research Center, Seattle, Washington; 3 Department of Biostatistics, University of Washington, Seattle; 4 Department of Pathology and Laboratory Medicine, University of Pennsylvania Perelman School of Medicine, Philadelphia; 5 Department of Medicine, Geisel School of Medicine at Dartmouth, Hanover, New Hampshire; 6 Department of Community and Family Medicine, Geisel School of Medicine at Dartmouth, Hanover, New Hampshire; 7 The Dartmouth Institute, Geisel School of Medicine at Dartmouth, Hanover, New Hampshire; 8 Department of Medical Informatics and Clinical Epidemiology, Oregon Health & Science University, Portland; 9 Department of Medicine, Oregon Health & Science University, Portland; 10 Pathology Associates, Clovis, California; 11 Department of Epidemiology, Norris Cotton Cancer Center, Geisel School of Medicine at Dartmouth, Hanover, New Hampshire; 12 Department of Biomedical Data Science, Norris Cotton Cancer Center, Geisel School of Medicine at Dartmouth, Hanover, New Hampshire; 13 Department of Family Medicine and of Public Health and Preventive Medicine, Oregon Health and Science University, Portland; 14 Department of Medicine, David Geffen School of Medicine, University of California, Los Angeles; 15 Department of Translational Research, Institut Curie, Paris, France

## Abstract

**Importance:**

Histopathologic criteria have limited diagnostic reliability for a range of cutaneous melanocytic lesions.

**Objective:**

To evaluate the association of second-opinion strategies by general pathologists and dermatopathologists with the overall reliability of diagnosis of difficult melanocytic lesions.

**Design, Setting, and Participants:**

This diagnostic study used samples from the Melanoma Pathology Study, which comprises 240 melanocytic lesion samples selected from a dermatopathology laboratory in Bellevue, Washington, and represents the full spectrum of lesions from common nevi to invasive melanoma. Five sets of 48 samples were evaluated independently by 187 US pathologists from July 15, 2013, through May 23, 2016. Data analysis was performed from April 2016 through November 2017.

**Main Outcomes and Measures:**

Accuracy of diagnosis, defined as concordance with an expert consensus diagnosis of 3 experienced pathologists, was assessed after applying 10 different second-opinion strategies.

**Results:**

Among the 187 US pathologists examining the 24 lesion samples, 113 were general pathologists (65 men [57.5%]; mean age at survey, 53.7 years [range, 33.0-79.0 years]) and 74 were dermatopathologists (49 men [66.2%]; mean age at survey, 46.4 years [range, 33.0-77.0 years]). Among the 8976 initial case interpretations, physicians desired second opinions for 3899 (43.4%), most often for interpretation of severely dysplastic nevi. The overall misclassification rate was highest when interpretations did not include second opinions and initial reviewers were all general pathologists lacking subspecialty training (52.8%; 95% CI, 51.3%-54.3%). When considering different second opinion strategies, the misclassification of melanocytic lesions was lowest when the first, second, and third consulting reviewers were subspecialty-trained dermatopathologists and when all lesions were subject to second opinions (36.7%; 95% CI, 33.1%-40.7%). When the second opinion strategies were compared with single interpretations without second opinions, the reductions in misclassification rates for some of the strategies were statistically significant, but none of the strategies eliminated diagnostic misclassification. Melanocytic lesions in the middle of the diagnostic spectrum had the highest misclassification rates (eg, moderately or severely dysplastic nevus, Spitz nevus, melanoma in situ, and pathologic stage [p]T1a invasive melanoma). Variability of in situ and thin invasive melanoma was relatively intractable to all examined strategies.

**Conclusions and Relevance:**

The results of this study suggest that second opinions rendered by dermatopathologists improve reliability of melanocytic lesion diagnosis. However, discordance among pathologists remained high.

## Key Points

**Question:**

For cutaneous melanocytic lesions that are difficult to diagnose, do second opinions rendered by pathologists who have board certification and/or fellowship training in dermatopathology improve overall reliability of diagnosis?

**Findings:**

In this diagnostic study of 240 melanocytic lesions from the Melanoma Pathology Study data set, misclassification of melanocytic lesions was lowest when first, second, and third consulting reviewers were subspecialty-trained dermatopathologists and when all lesions were subject to second opinions; misclassification was highest when reviewers were all general pathologists lacking subspecialty training. Variability of in situ and thin invasive melanoma was relatively intractable to all examined strategies.

**Meaning:**

The findings suggest that second opinions rendered by dermatopathologists improve overall reliability of diagnosis of melanocytic lesions but do not eliminate or substantially reduce misclassification.

## Teaser

This diagnostic study evaluates whether fellowship training and board certification of physicians in dermatopathology affect the diagnostic reliability of second-opinion strategies for difficult-to-diagnose cutaneous melanocytic lesions.

## Introduction

Second opinions in medicine are used to reach consensus about the diagnosis and management of patients with the goal of improving patient care. Second opinions may include (1) a brief verbal or written opinion, (2) a formal written review often in compliance with institutional policies, and (3) an independent detailed review by a recognized expert. Given the high rates of diagnostic disagreement for certain skin biopsy samples, especially those of challenging melanocytic proliferations,^1^ mandatory second opinions have been advocated.^2^ Because nearly 5 million adults are treated for skin cancer annually, with mean treatment costs of more than $8 billion each year, ensuring quality diagnoses is imperative.^3^ With increases in the Medicare population over time, an increased number of skin cancer procedures have been performed each year.^4^ Quantifying the value of mandatory second-opinion review, however, is essential before instituting mandates.

Although studies suggest that second opinions reduce disagreement rates,^5^ the optimal selection of cases for review and the best practices for them are unknown. With millions of biopsies performed annually,^6^ second opinions for all skin pathology cases is not feasible.^6^ Therefore, it is a priority to identify strategies and policies for second opinions that both are practical and can best improve clinical care.

In a recent survey of 207 pathologists,^7^ most respondents perceived that second opinions increase accuracy (78%) and protect them from malpractice lawsuits (62%). Although a small number of respondents reported that second opinions are mandated by laboratories for lesions such as melanoma in situ (26%) and invasive melanoma (30%), pathologists desire second opinions most often for melanocytic tumors of uncertain malignant potential (85%) and atypical Spitz tumors (88%).^7^

The present study follows recent work^8^ that reported an association between fellowship training and board certification in dermatopathology and improved diagnostic reliability. Given the importance of accurate diagnosis and the clinical consequences of errors, the present study compared different second-opinion strategies using data from individual interpretations made by pathologists, including general pathologists and board-certified and/or fellowship-trained dermatopathologists. For different strategies, we assessed rates of overinterpretation and underinterpretation and overall misclassification relative to a consensus reference diagnosis.

## Methods

### Test Set Cases and Consensus Reference Diagnoses

This diagnostic study used samples from the Melanoma Pathology Study, details for which are reported elsewhere.^1,9^ The institutional review board at the University of Washington, Seattle, approved all study procedures, and all pathologists provided informed consent electronically. Data analysis was performed from April 2016 to November 2017. This study followed the Standards for Reporting of Diagnostic Accuracy (STARD) reporting guideline for diagnostic studies.

In brief, skin specimens, represented by 1 slide per case, were selected from a dermatopathology laboratory in Bellevue, Washington. Three experienced pathologists (M.W.P, D.E.E., and R.L.B.) interpreted each case independently before arriving at a consensus reference diagnosis by modified Delphi technique.^10^ Reference diagnoses were categorized using the Melanocytic Pathology Assessment Tool and Hierarchy for Diagnosis (MPATH-Dx) schema.^11^ The final 240 Melanoma Pathology Study samples were randomly assigned to 5 test sets stratified by patient age (20-49 years, 50-64 years, and ≥65 years) and by MPATH-Dx category, with example diagnostic terms and suggested treatment as follows: (I) common nevus or mildly dysplastic nevus, with no treatment required (10%); (II) moderately dysplastic nevus or Spitz nevus, with suggested reexcision with less than 5-mm margins (15%); (III) severely dysplastic nevus or melanoma in situ, with reexcision with 5-mm to less than 1-cm margins (25%); (IV) thin invasive melanoma (pathologic stage [p]T1a), with wide excision with at least 1-cm margins (24%); and (V) thicker invasive melanoma (pT1b or greater), with wide excision with at least 1-cm margins and consideration of additional diagnostic workup or adjuvant therapy (25%). Cases of MPATH-Dx categories III through V were oversampled.^1^

### Participating Pathologists

Pathologists from 10 states were invited to participate (California, Connecticut, Hawaii, Iowa, Kentucky, Louisiana, New Jersey, New Mexico, Utah, and Washington), as described elsewhere.^1,12^ Pathologists were eligible if they had interpreted skin biopsies in the previous year, planned to continue interpreting them for the following 2 years, and were not in resident or fellowship training. A web-based survey queried participants about demographics, clinical practices, use of second opinions in their practice, and interpretive experience.^7^ General pathologists (no board certification or fellowship training in dermatopathology) and dermatopathologists (board certification and/or fellowship training in dermatopathology) were defined based on survey responses. Of 301 eligible pathologists, 207 (68.8%) were enrolled and 187 (62.1%) completed independent phase 1 interpretations. More details about the recruitment and follow-up of participants can be reviewed elsewhere.^1^

Participants were randomized to examine 1 of 5 sets of 48 samples, recording their interpretations using an online MPATH-Dx tool from July 15, 2013, through May 23, 2016. For each sample, participants indicated whether a second opinion was personally desired before finalizing the diagnosis, whether it would be required by their laboratory policies for the particular diagnosis, or both.

### Strategies for Obtaining Second Opinions

**Figure 1.**
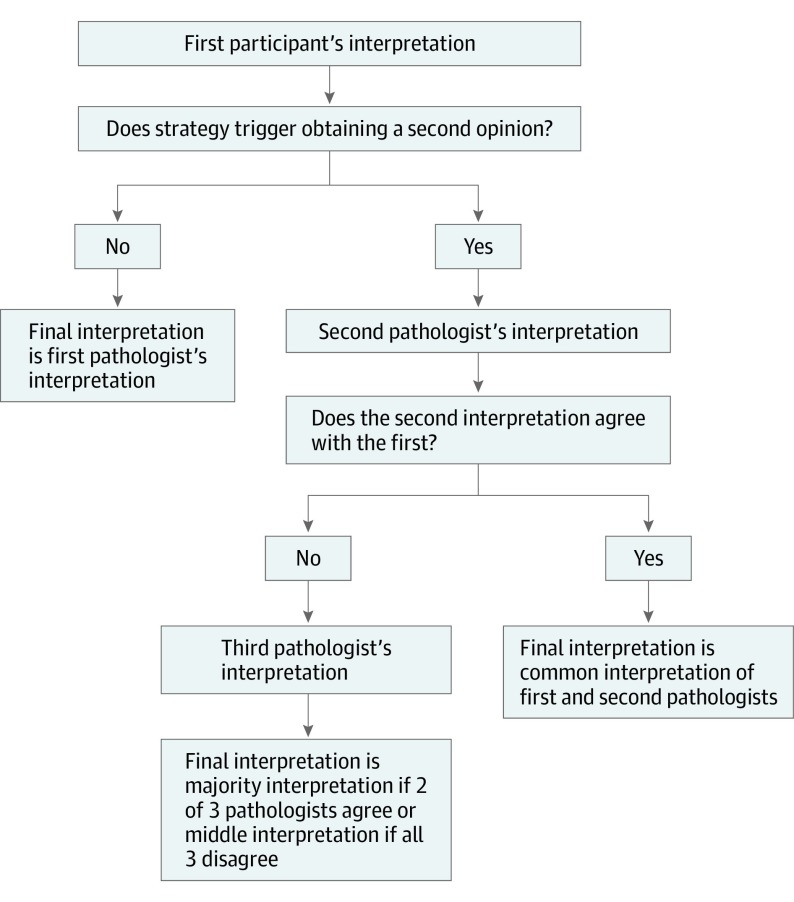
Determination of Final Skin Biopsy Sample Interpretation When Considering Different Strategies for Obtaining a Second Opinion Strategy for obtaining a second opinion was based on the first pathologist’s interpretation, on self-reports of second opinion desired or mandated, or on the level of dermatopathology training. When considering different strategies for obtaining a second opinion, up to 3 pathologists may be needed to obtain a final interpretation. Data comprised 11 603 808 observations, each involving 3 independent pathologists’ interpretations of a skin biopsy sample, and are derived from findings of 187 single pathologists interpreting 48 samples each in 5 test sets.

Interpretations that incorporated second opinions were defined by considering each possible pair of participants interpreting a case independently and, when 2 interpretations disagreed, resolution by a third, independent interpretation. Resolution was achieved by assigning the sample to the MPATH-Dx diagnostic category identified by 2 of 3 participants or, if all 3 physicians disagreed, assigning the middle diagnosis (Figure 1). All possible paired combinations were considered because we sought to examine, on average, how second opinions were associated with accuracy. We evaluated 10 strategies for obtaining a second opinion, detailed in eTable 1 in the Supplement. The first 6 strategies were based on the initial diagnostic interpretation, whereas the last 4 strategies were based on the primary and consulting physicians’ professional qualifications.

### Statistical Analysis

We calculated rates of overinterpretation, underinterpretation, and overall misclassification relative to reference consensus diagnoses. Overinterpretation was defined as classification at a more severe MPATH-Dx category, underinterpretation as classification at a lower category, and misclassification as either overinterpretation or underinterpretation. For implementation, we combined the independent interpretations of the study participants (Figure 1). When a second opinion agreed with the initial diagnosis, that shared diagnosis became the final diagnosis. When a second opinion disagreed with the initial diagnosis, a third opinion was obtained. The final diagnosis was then either the majority or middle diagnosis (in cases in which all 3 opinions differed). The percentage of cases in which a second and third opinion was required for each of the strategies is shown in eTable 2 and eTable 3 in the Supplement.

Second-opinion strategies were implemented by creating ordered data records of interpretations for every case and for every 3 participants who interpreted the case, and the majority or middle interpretation was selected as the final assessment. This approach was analytically equivalent to that described in the preceding paragraph and correctly weighted interpretations of cases in which the second opinion agreed and disagreed with the initial interpretation. The approach produced 11 603 808 data records of 3 independent interpretations of the same case: 39 pathologists interpreted the 48 cases in test set A, 36 interpreted test set B, 38 interpreted test set C, 36 interpreted test set D, and 38 interpreted test set E, yielding a total of 48 × (39 × 38 × 37 + 36 × 35 × 34 + 38 × 37 × 36 + 36 × 35 × 34 + 38 × 37 × 36) = 11 603 808 ordered triple interpretations.

The 95% CIs for the overinterpretation and underinterpretation and overall misclassification rates used percentiles of the bootstrap distribution of each rate in which resampling of participants was performed 1000 times. Second-opinion interpretations that included the same participant for second or third interpretations were discarded from the bootstrapped estimates. The 2-sided *P* values for the Wald test of a difference in rates between the single participant and second-opinion strategies were derived from the bootstrap SE of the difference in rates.

A secondary analysis examined agreement rather than accuracy. To quantify agreement between single interpretations, rates were calculated from a simple cross-tabulation of all pairs of single interpretations of the same cases; because the computational burden of this approach for the 11 603 808 assessments involving second opinions was not tenable, we used a different approach. We paired all triple readings of each case with a random permutation of those triple readings for that case resulting in a mean of 48 350 triple reading pairs per case. After excluding triple reading pairs that involved the same reader in both readings, we combined data for all cases and calculated agreement statistics from the cross-tabulation. We replicated this procedure 1000 times and report mean agreement and κ statistics. All analyses were conducted with Stata, version 14 (StataCorp LLC).

## Results

Of 187 pathologists participating in this study, 113 were general pathologists (65 men [57.5%]; mean [range] age at survey, 53.7 [33.0-79.0] years) and 74 were dermatopathologists (49 men [66.2%]; mean [range] age at survey, 46.4 [33.0-77.0] years). Board-certified and/or fellowship-trained dermatopathologists were more likely than other participating pathologists to be affiliated with an academic medical center. The dermatopathologists reported fewer years of experience interpreting melanocytic skin lesions, a higher proportion of caseload involving interpretations of these lesions, and being held by their peers as experts in their interpretation (Table 1).

**Table 1.** Demographic and Experience Characteristics of Pathologists by Dermatopathology Training

Characteristic	Pathologist Type, No. (%)^a^		
	Total (N = 187)	General Pathologist (n = 113)^b^	Dermatopathologist (n = 74)^c^
Age at survey, mean (range), y	NA	53.7 (33.0-79.0)	46.4 (33.0-77.0)
Sex			
Female	73 (39.0)	48 (42.5)	25 (33.8)
Male	114 (61.0)	65 (57.5)	49 (66.2)
Academic medical center affiliation			
No	134 (71.7)	96 (85.0)	38 (51.4)
Adjunct or affiliation	34 (18.2)	14 (12.4)	20 (27.0)
Primary appointment	19 (10.2)	3 (2.7)	16 (21.6)
Experience interpreting melanocytic skin lesions, y			
0-4	29 (15.5)	13 (11.5)	16 (21.6)
5-9	45 (24.1)	20 (17.7)	25 (33.8)
10-19	57 (30.5)	33 (29.2)	24 (32.4)
≥20	56 (30.0)	47 (41.6)	9 (12.2)
Clinical work interpreting melanocytic skin lesions, % of total clinical caseload			
0-9	79 (42.2)	75 (66.4)	4 (5.4)
10-24	72 (38.5)	34 (30.1)	38 (51.4)
25-49	28 (15.0)	4 (3.5)	24 (32.4)
≥50	8 (4.3)	0	8 (10.8)
Do your colleagues consider you an expert in melanocytic skin lesions?			
No	108 (57.8)	98 (86.7)	10 (13.5)
Yes	79 (42.2)	15 (13.3)	64 (86.5)

Abbreviation: NA, not available.

^a^ Percentages may not sum to 100% because of rounding.

^b^ General pathologist defined as having no fellowship training or board certification.

^c^ Dermatopathologist defined as having fellowship training and/or board certification.

**Figure 2.**
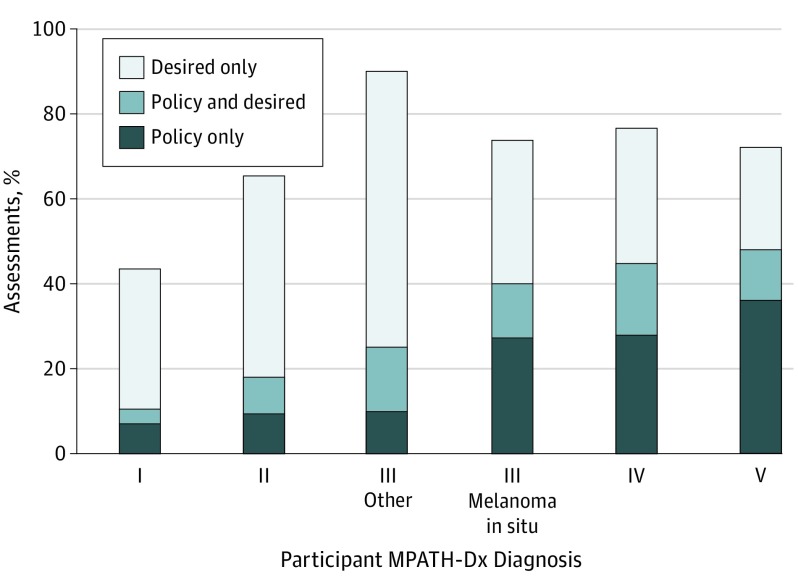
Second Opinion Desired and/or Required by Policy Participant responses to second-opinion question shown for each interpretation provided on that case (N = 8976). Wording of the Melanocytic Pathology Assessment Tool and Hierarchy for Diagnosis (MPATH-Dx) question was as follows: “Would you ask for a second pathologist's opinion of this case before finalizing the report? (assume a second pathologist is available; check all that apply).” Answers included (1) no; (2) yes, because it is our policy to get a second opinion on cases with this diagnosis; and (3) yes, because I would want a second pathologist’s opinion for diagnostic reasons (eg, the diagnosis is challenging, borderline, or uncertain). Physician diagnoses are shown for MPATH-Dx categories I to V.

Among the 8976 initial case interpretations, physicians desired second opinions for 43.4% (n = 3899), most often for interpretation of severely dysplastic nevi and least often for interpretations of benign lesions (Figure 2). Second opinions were desired more often than mandated by policy except for interpretations of class V invasive melanoma. Physicians’ report of policy-mandated second opinions increased from MPATH-Dx class I (10.5%) through class II (18.1%), class III other (25.0%), class III melanoma in situ (40.1%), class IV (44.6%), and class V (47.8%).

### Strategies 1 Through 6—Based on Initial Interpretation of the Case

The highest misclassification rate within diagnostic categories after a single interpretation was for moderately dysplastic nevus (75.3%; 95% CI, 73.2%-77.5%), followed by severely dysplastic nevus or melanoma in situ (59.6%; 95% CI, 57.4%-61.8%), invasive pT1a melanoma (57.2%; 95% CI, 54.9%-59.7%), at least invasive pT1b melanoma (27.9%; 95% CI, 26.0%-29.9%), and benign nevi (7.8%; 95% CI, 6.2%-9.4%) (Table 2). The overall misclassification rate for a single interpretation (47.9%; 95% CI, 46.7%-49.1%) was the performance reference for second-opinion strategies 1 through 6. The lowest misclassification rate resulted when second opinions were applied to all skin biopsy samples (strategy 1: 44.8%; 95% CI, 42.5%-47.1%; *P* < .001). With strategy 1, the rates for overinterpretation decreased from 6.3% (95% CI, 5.7%-6.9%) to 3.1% (95% CI, 2.4%-4.0%) and were unchanged for underinterpretation (41.6% [95% CI, 40.3%-43.0%] to 41.7% [95% CI, 39.2%-44.2%]).

**Table 2.** Six Second-Opinion Strategies Based on Initial Interpretation

Strategy	Rate, % (95% CI)^a^					Overall	*P* Value
	Melanoma Pathology Study Reference Consensus Diagnosis						
	Class I, Benign	Class II, Moderately Dysplastic	Class III, Severely Dysplastic/Melanoma In Situ	Class IV, pT1a Melanoma	Class V, At Least pT1b Melanoma		
**Single Interpretation**							
Overinterpretation	7.8 (6.2-9.4)	12.5 (11.0-14.1)	5.5 (4.6-6.4)	9.1 (7.8-10.5)	0	6.3 (5.7-6.9)	NA
Underinterpretation	0	62.8 (60.2-65.4)	54.1 (51.5-56.7)	48.1 (46.0-50.2)	27.9 (26.0-29.9)	41.6 (40.3-43.0)	NA
Misclassification	7.8 (6.2-9.4)	75.3 (73.2-77.5)	59.6 (57.4-61.8)	57.2 (54.9-59.7)	27.9 (26.0-29.9)	47.9 (46.7-49.1)	NA
**Strategy 1, Second Opinion Applied to All Cases**							
Overinterpretation	3.3 (1.6-5.7)	7.2 (4.9-10.0)	2.2 (1.3-3.5)	4.7 (3.1-6.5)	0	3.1 (2.4-4.0)	NA
Underinterpretation	0	65.8 (59.2-71.3)	54.9 (49.8-61.4)	47.7 (43.9-51.6)	26.0 (21.6-31.2)	41.7 (39.2-44.2)	NA
Misclassification	3.3 (1.6-5.7)	73.0 (67.8-77.5)	57.1 (52.4-63.3)	52.4 (48.4-56.5)	26.0 (21.6-31.2)	44.8 (42.5-47.1)	<.001
**Strategy 2, Criterion for Obtaining Second Opinion Based on Initial Diagnosis, Initial T1b or Greater**							
Overinterpretation	7.5 (4.8-10.8)	12.0 (9.4-14.5)	5.3 (4.0-6.6)	3.4 (2.2-4.8)	0	4.7 (3.9-5.6)	NA
Underinterpretation	0	63.0 (57.9-67.2)	53.8 (49.8-58.9)	49.2 (46.2-52.2)	33.6 (29.0-38.8)	43.3 (41.2-45.4)	NA
Misclassification	7.5 (4.8-10.8)	74.9 (71.3-78.2)	59.1 (55.5-63.5)	52.6 (49.4-55.7)	33.6 (29.0-38.8)	48.0 (46.1-49.9)	.92
**Strategy 3, Initial T1a or T1b or Greater**							
Overinterpretation	7.2 (4.6-10.6)	11.0 (8.5-13.7)	1.5 (0.9-2.5)	4.3 (2.8-6.0)	0	3.8 (3.1-4.7)	NA
Underinterpretation	0	63.4 (58.3-67.7)	54.9 (50.8-60.1)	57.4 (54.0-60.8)	29.9 (25.6-34.9)	44.7 (42.4-46.9)	NA
Misclassification	7.2 (4.6-10.6)	74.4 (70.8-77.8)	56.4 (52.6-61.4)	61.8 (58.1-65.3)	29.9 (25.6-34.9)	48.5 (46.5-50.6)	.44
**Strategy 4, Initial Melanoma In Situ, T1a, or T1b or Greater**							
Overinterpretation	7.2 (4.6-10.5)	9.9 (7.5-12.4)	1.7 (1.0-2.7)	4.4 (2.9-6.1)	0	3.7 (3.0-4.6)	NA
Underinterpretation	0	63.9 (59.0-68.2)	58.1 (53.7-63.8)	54.4 (50.9-58.0)	29.7 (25.4-34.9)	44.8 (42.6-47.1)	NA
Misclassification	7.2 (4.6-10.5)	73.8 (70.0-77.2)	59.8 (55.6-65.3)	58.9 (55.1-62.6)	29.7 (25.4-34.9)	48.5 (46.4-50.6)	.44
**Strategy 5, Second Opinion Obtained Only When Desired**							
Overinterpretation	4.4 (2.4-7.0)	8.2 (5.9-11.0)	3.9 (2.6-5.3)	6.9 (4.8-9.1)	0	4.3 (3.5-5.2)	NA
Underinterpretation	0	66.1 (60.4-70.9)	54.7 (50.3-60.7)	45.8 (42.1-49.5)	25.9 (21.9-30.6)	41.2 (38.8-43.6)	NA
Misclassification	4.4 (2.4-7.0)	74.3 (69.8-78.2)	58.6 (54.5-64.2)	52.7 (48.9-56.6)	25.9 (21.9-30.6)	45.5 (43.5-47.5)	.001
**Strategy 6, Second Opinion Obtained When Desired or Required by Policy**							
Overinterpretation	4.2 (2.2-6.8)	7.1 (4.8-10.0)	2.8 (1.8-4.1)	5.4 (3.5-7.3)	0	3.5 (2.7-4.5)	NA
Underinterpretation	0	66.6 (60.8-71.5)	55.5 (50.7-61.6)	46.8 (43.0-50.6)	26.4 (21.9-31.4)	41.8 (39.4-44.3)	NA
Misclassification	4.2 (2.2-6.8)	73.7 (69.0-77.8)	58.3 (53.8-64.1)	52.1 (48.2-56.0)	26.4 (21.9-31.4)	45.3 (43.2-47.4)	.001

Abbreviation: NA, not applicable.

^a^ Overinterpretation, underinterpretation, and misclassification rates of single interpretation compared with a reference consensus diagnosis.

For strategies 2 through 4 (second opinions obtained when initial interpretations were melanoma in situ or invasive melanoma), the overall misclassification rates were similar to the single interpretation misclassification rate. For strategies 5 and 6 (physician noted that they desire a second opinion [45.5%; 95% CI, 43.5%-47.5%] or second opinions would be desired or required in their practice for this type of case [45.3%; 95% CI, 43.2%-47.4%], respectively), misclassification rates were decreased compared with the single interpretation rate (47.9%; 95% CI, 46.7%-49.1%) (*P* < .001).

### Strategies 7 Through 10—Based on Primary and Consulting Physicians’ Experience

When the initial physician was neither board certified nor fellowship trained in dermatopathology (ie, a general pathologist), the highest misclassification rate after single interpretation was for moderately dysplastic nevi (79.3%; 95% CI, 77.0%-81.6%), followed by severely dysplastic nevi or melanoma in situ (67.1%; 64.7%-69.7%), invasive pT1a melanomas (66.2%; 95% CI, 63.3%-69.5%), at least invasive pT1b melanomas (29.4%; 95% CI, 26.9%-31.9%), and benign melanocytic lesions (6.5%; 95% CI, 4.7%-8.2%) (Table 3). When the initial physician was board certified and/or fellowship trained (ie, a dermatopathologist), the misclassification rates after single interpretation decreased for moderately dysplastic nevi (69.3%; 95% CI, 65.5%-73.2%), severely dysplastic nevi or melanoma in situ (48.1%; 44.8%-51.0%), invasive pT1a melanomas (43.5%; 95% CI, 40.3%-46.6%), and at least pT1b melanomas (25.7%; 95% CI, 22.8%-28.5%).

**Table 3.** Four Second-Opinion Strategies Based on Primary and Consulting Pathologists’ Experience

Strategy	Rate, % (95% CI)^a^					Overall	*P* Value^b^
	Melanoma Pathology Study Reference Consensus Diagnosis						
	Class I, Benign	Class II, Moderately Dysplastic	Class III, Severely Dysplastic/Melanoma In Situ	Class IV, pT1a Melanoma	Class V, At Least pT1b Melanoma		
**Single Interpretation for General Pathologists**^**c**^								
Overinterpretation	6.5 (4.7-8.2)	10.8 (9.0-12.4)	4.6 (3.5-5.6)	11.1 (9.3-13.1)	0	6.1 (5.4-6.9)	NA
Underinterpretation	0	68.6 (65.6-71.5)	62.5 (59.5-65.6)	55.1 (52.4-57.9)	29.4 (26.9-31.9)	46.7 (44.9-48.3)	NA
Misclassification	6.5 (4.7-8.2)	79.3 (77.0-81.6)	67.1 (64.7-69.7)	66.2 (63.3-69.5)	29.4 (26.9-31.9)	52.8 (51.3-54.3)	NA
**Strategy 7, Second and Third General Pathologists**^**c**^								
Overinterpretation	2.0 (0.7-3.9)	5.8 (3.0-9.3)	2.1 (0.8-3.5)	5.6 (3.1-8.5)	0	2.9 (2.0-4.0)	NA
Underinterpretation	0	72.2 (62.5-79.9)	63.6 (56.3-73.6)	56.0 (50.9-60.7)	27.1 (21.2-33.6)	47.1 (44.2-50.0)	NA
Misclassification	2.0 (0.7-3.9)	78.0 (71.0-83.9)	65.7 (59.1-74.9)	61.6 (55.8-67.3)	27.1 (21.2-33.6)	50.1 (47.4-52.7)	.01
**Strategy 8, Second General Pathologists and Third Dermatopathologists**^**c**^^,^^**d**^								
Overinterpretation	2.7 (1.4-4.4)	6.9 (4.6-9.5)	2.2 (1.2-3.5)	4.8 (3.0-6.8)	0	3.0 (2.3-3.8)	NA
Underinterpretation	0	67.3 (60.9-73.0)	57.5 (52.2-64.2)	49.2 (45.3-53.0)	26.0 (21.4-31.3)	43.0 (40.6-45.2)	NA
Misclassification	2.7 (1.4-4.4)	74.2 (68.7-78.9)	59.7 (54.9-66.0)	54.0 (49.8-58.2)	26.0 (21.4-31.3)	46.0 (43.7-48.2)	.001
**Strategy 9, Second and Third Dermatopathologists**^**d**^								
Overinterpretation	4.4 (1.8-8.2)	8.3 (5.2-11.9)	2.4 (1.3-3.7)	4.1 (2.4-6.1)	0	3.3 (2.4-4.4)	NA
Underinterpretation	0	60.9 (54.2-66.8)	47.3 (42.3-53.0)	41.7 (38.1-45.7)	25.3 (19.9-31.7)	37.4 (35.0-40.0)	NA
Misclassification	4.4 (1.8-8.2)	69.1 (63.0-74.4)	49.7 (45.1-54.7)	45.8 (41.9-50.2)	25.3 (19.9-31.7)	40.7 (38.4-43.1)	.001
**Single Interpretation for Dermatopathologists**^**d**^								
Overinterpretation	9.7 (6.5-12.7)	15.2 (12.1-18.2)	6.9 (5.4-8.3)	6.1 (4.4-7.5)	0	6.5 (5.5-7.4)	NA
Underinterpretation	0	54.1 (49.7-58.9)	41.2 (37.6-44.6)	37.4 (34.8-40.1)	25.7 (22.8-28.5)	34.0 (32.0-35.9)	NA
Misclassification	9.7 (6.5-12.7)	69.3 (65.5-73.2)	48.1 (44.8-51.0)	43.5 (40.3-46.6)	25.7 (22.8-28.5)	40.5 (38.8-42.1)	NA
**Strategy 10, Second and Third Dermatopathologists**^**d**^								
Overinterpretation	7.2 (1.6-16.1)	9.9 (4.6-16.2)	2.6 (1.1-4.6)	3.6 (1.5-6.0)	0	3.8 (2.2-6.1)	NA
Underinterpretation	0	55.1 (45.6-63.8)	39.2 (33.3-45.9)	34.9 (29.6-40.4)	25.4 (18.2-35.5)	33.0 (29.0-37.5)	NA
Misclassification	7.2 (1.6-16.1)	65.0 (57.4-72.3)	41.8 (35.9-48.4)	38.5 (33.4-44.5)	25.4 (18.2-35.5)	36.7 (33.1-40.7)	.01

Abbreviation: NA, not applicable.

^a^ Overinterpretation, underinterpretation, and misclassification rates of single interpretation compared with a reference consensus diagnosis.

^b^ *P* values are based on a Wald test for the difference in overall misclassification rates between the second-opinion strategy and the single pathologist interpretation. The test statistic uses the bootstrap SE of the difference in rates.

^c^ General pathologist defined as having no fellowship training or board certification.

^d^ Dermatopathologist defined as having fellowship training and/or board certification.

The overall misclassification rate for single interpretations by general pathologists was 52.8% (95% CI, 51.3%-54.3%). For second-opinion strategies 7 through 9, misclassification rates were reduced, with the rate for strategy 9 (both second and third opinions obtained from dermatopathologists) being lowest (40.7%; 95% CI, 38.4%-43.1%; *P* < .001). With strategy 9, the rates for overinterpretation decreased from 6.1% (95% CI, 5.4%-6.9%) to 3.3% (95% CI, 2.4%-4.4%) and decreased for underinterpretation from 46.7% (95% CI, 44.9%-48.3%) to 37.4% (95% CI, 35.0%-40.0%) (Table 3).

For strategy 10, all of the interpretations (initial, second, and, if needed, third opinions) were obtained from a dermatopathologist, giving an overall misclassification rate for single interpretations of 40.5% (95% CI, 38.8%-42.1%). For second-opinion strategy 10 (second and third opinions by dermatopathologists), the rate was reduced to 36.7% (95% CI, 33.1%-40.7%) (*P* = .01). With this strategy, the rates for overinterpretation decreased from 6.5% (95% CI, 5.5%-7.4%) to 3.8% (95% CI, 2.2%-6.1%) and rates for underinterpretation were 34.0% (32.0%-35.9%) and 33.0% (95% CI, 29.0%-37.5%), respectively.

### Agreement Among Interpretations

Our secondary analysis evaluated agreement among interpretations. The mean between physician pairwise rate for single interpretations of a case was 54.8%, whereas the corresponding rate for interpretations that included second opinions was 64.3%. The corresponding κ statistics were 0.42 and 0.55, indicating, at most, moderate levels of agreement.

## Discussion

Studies from the past several decades have uncovered limited diagnostic reliability of histopathologic criteria in a clinically important segment of melanocytic lesion categories.^13,14,15,16^ The largest controlled study to date found that limitations predominantly affect the spectrum from dysplastic nevus, through melanoma in situ, and pT1a melanoma,^1,17^ corresponding to MPATH-Dx classes II through IV.^11^ A population-based analysis estimated that the spectrum of melanocytic skin biopsies affected by poor diagnostic reliability represents approximately 15% of all melanocytic lesions subject to biopsy.^18^

The diagnostic process derives from physician assessment of phenotypic alterations along criteria scales, among which are size (ie, diameter), symmetry, circumscription, pagetoid scatter, nuclear atypia, and maturation effects. These attributes constitute more or less continuous scales of variability, with no objective break points. Thus, the diagnostic process for current classifications devolves to pathologist-dependent threshold setting, in which the pathologist determines that sufficient abnormalities do or do not exist along the scales for definitive classification. When histopathologic imagery is parsed to increasingly fine detail, the limits of subjective histopathologic interpretation are reflected in escalating rates of diagnostic discordance.

Our study found that second-opinion strategies of primary and consulting pathologists in which first, second, and third reviewers were fellowship-trained and/or board-certified dermatopathologists yielded the lowest misclassification rates. Conversely, the highest misclassification rates occurred with general pathologists. However, the absolute magnitude of calculated differences, while statistically significant, was modest; improvement in rates for overdiagnosis and underdiagnosis and for misclassification ranged from less than 5% to 10% for most comparisons with single physician results. Misclassification rates were also improved with the strategy of requiring second opinions for all lesions, but this protocol is not feasible and likely not cost-effective in general practice. Among melanomas in situ and thin invasive (pT1a per the American Joint Committee on Cancer) melanomas, only small improvements in misclassification rates occurred when second and third opinions were factored into the diagnostic algorithm. Of note, it is this portion of the melanocytic lesion spectrum for which general policy mandates are often in place. The same intractability to second-opinion strategies applies to gradations of melanocytic dysplasia. An evaluation of strategies for breast histopathologic examination^19^ reported that second opinions improved diagnostic agreement for benign and invasive lesions but less so for lesions with intermediate atypia.

The observation that none of the second-opinion strategies eliminated or substantially reduced diagnostic variability underscores the need to advance the basic science of melanocytic proliferations and implement alternative diagnostic technologies. At present, simplifying current classification schemes to eliminate clinically irrelevant distinctions could be considered. This approach was adopted in the recent World Health Organization classification,^20^ which combined the categories of mild dysplasia and banal nevi, yielding a 2-tier categorization of low- and high-grade dysplasia.

### Limitations

Among the limitations of this study, all interpretations were by completely independent reviewers, with blinding of the primary and consulting assessments and no consensus discussion. A consultant in the real world is usually cognizant of the primary reviewer’s diagnosis and not blinded; however, studying differing second-opinion strategies in clinical practice would not be feasible because of complexity, cost, liability issues, privacy concerns, and/or regulatory constraints. Our approach provided the most feasible current estimates of how differing second-opinion strategies could affect diagnostic fidelity. The composition of Melanoma Pathology Study cases is different from clinical practice because case selection was intentionally weighted to achieve statistical power for lesions in the intermediate sector of melanocytic proliferations (MPATH-Dx classes II-IV). Because of this case composition, we provided results by the individual MPATH-Dx class. Furthermore, we did not address the role of advanced clinical experience, the nonequivalency of consultants, and the corresponding effects of such factors on accuracy in histologic diagnosis.

## Conclusions

This study’s results suggest that there is uncertainty in the interpretations of some proportion of difficult melanocytic lesions by both primary observers and the consultants who render second opinions. However, a second opinion contributed by an expert may facilitate consensus about the appropriate management of a difficult melanocytic lesion.

## Notes

Supplement. **eTable 1.** Summary of Strategies Evaluated for Obtaining Second Opinion **eTable 2.** Six Second Opinion Strategies Based on Initial Interpretation: Percentage Requiring Second and Third Opinions **eTable 3.** Four Second Opinion Strategies Based on Primary and Consulting Pathologists’ Experience: Percentage Requiring Third Opinion
