# Supplementary material for: Assessment of Second-Opinion Strategies for Diagnoses of Cutaneous Melanocytic Lesions
Source: JAMA Netw Open. 2019 Oct 11;2(10):e1912597. doi: 10.1001/jamanetworkopen.2019.12597 (PMC6804025; doi:10.1001/jamanetworkopen.2019.12597)
Supplement: Supplement. — eTable 1. Summary of Strategies Evaluated for Obtaining Second Opinion eTable 2. Six Second Opinion Strategies Based on Initial Interpretation: Percentage Requiring Second and Third Opinions eTable 3. Four Second Opinion Strategies Based on Primary and Consulting Pathologists’ Experience: Percentage Requiring Third Opinion [file jamanetwopen-2-e1912597-s001.pdf]

# Supplementary Online Content

Piepkorn MW, Longton GM, Reisch LM, et al. Assessment of second-opinion strategies for diagnoses of cutaneous melanocytic lesions. *JAMA Netw Open*. 2019;2(10):e1912597. doi:10.1001/jamanetworkopen.2019.12597

**eTable 1.** Summary of Strategies Evaluated for Obtaining Second Opinion

**eTable 2.** Six Second Opinion Strategies Based on Initial Interpretation: Percentage Requiring Second and Third Opinions

**eTable 3.** Four Second Opinion Strategies Based on Primary and Consulting Pathologists' Experience: Percentage Requiring Third Opinions

This supplementary material has been provided by the authors to give readers additional information about their work.

© 2019 Piepkorn MW et al. *JAMA Network Open*

| **eTable 1**. Summary of Strategies Evaluated for Obtaining Second Opinion |
|---|
| **Strategy 1. Second opinion is applied to all skin biopsy cases** |
| **Second opinion obtained only if:** |
| Strategy 2. initial interpretations considered pT1b melanoma or greater (M-Path category V) |
| Strategy 3. initial interpretations considered melanoma pT1a or pT1b or greater (M-Path category IV or V) |
| Strategy 4. initial interpretations considered melanoma in situ or invasive melanoma (M-Path categories III MIS only, IV and V) |
| Strategy 5. initial pathologist desires a second opinion |
| Strategy 6. initial pathologist desires a second opinion or if it would be required by policy |
| **Evaluation restricted to cases read by initial pathologists with no board certification or fellowship training in dermatopathology (i.e., general pathologist):** |
| Strategy 7. with 2nd and 3rd brought in from another pathologist with no board certification or fellowship training in dermatopathology |
| Strategy 8. with 2nd brought in from another pathologist with no board certification or fellowship training in dermatopathology, and 3rd brought in from a board-certified and/or fellowship trained dermatopathologist |
| Strategy 9. with 2nd and 3rd brought in from a dermatopathologist with board certification and/or fellowship training in dermatopathology |
| **Evaluation restricted to cases read by initial dermatopathologists with board certification and/or fellowship training in dermatopathology:** |
| Strategy 10. with 2nd and 3rd brought in from a dermatopathologist with board certification and/or fellowship training in dermatopathology |

© 2019 Piepkorn MW et al. *JAMA Network Open*

**eTable 2**. Six Second Opinion Strategies Based on Initial Interpretation: Percentage Requiring Second and Third Opinions

| Strategy | Rate, % | | | | | |
|---|---|---|---|---|---|---|
| | M-Path Reference Consensus Diagnosis | | | | | |
| | Benign (I) | Moderately Dysplastic (II) | Severely Dysplastic/MIS (III) | pT1a melanoma (IV) | pT1b melanoma or greater (V) | Overall |
| **SECOND OPINION APPLIED TO ALL CASES**<br>**Strategy 1** | | | | | | |
| % requiring 2nd opinion | 100.0 | 100.0 | 100.0 | 100.0 | 100.0 | 100.00 |
| % requiring 3rd opinion | 13.4 | 45.6 | 56.6 | 59.4 | 33.5 | 45.2 |
| **CRITERION FOR OBTAINING SECOND OPINION BASED ON INITIAL DIAGNOSIS**<br>**Strategy 2. (Initial t1b)** | | | | | | |
| % requiring 2nd opinion | 0.1 | 0.8 | 0.5 | 9.1 | 72.1 | 20.8 |
| % requiring 3rd opinion | 0.1 | 0.8 | 0.5 | 7.0 | 12.8 | 5.2 |
| **Strategy 3. (Initial t1a or t1b)** | | | | | | |
| % requiring 2nd opinion | 0.4 | 2.8 | 5.5 | 51.9 | 86.2 | 36.3 |
| % requiring 3rd opinion | 0.4 | 2.5 | 4.9 | 27.9 | 21.8 | 13.9 |

© 2019 Piepkorn MW et al. *JAMA Network Open*

| Strategy | Rate, % | | | | | |
|---|---|---|---|---|---|---|
| | M-Path Reference Consensus Diagnosis | | | | | |
| | Benign (I) | Moderately Dysplastic (II) | Severely Dysplastic/MIS (III) | pT1a melanoma (IV) | pT1b melanoma or greater (V) | Overall |
| **Strategy 4. (initial MIS, t1a or t1b)** | | | | | | |
| % requiring 2nd opinion | 0.5 | 4.6 | 27.5 | 69.1 | 87.1 | 46.5 |
| % requiring 3rd opinion | 0.5 | 4.0 | 12.9 | 36.2 | 22.6 | 18.4 |
| **SECOND OPINION ONLY OBTAINED FOR CASES WHEN DESIRED OR REQUIRED BY POLICY OR BOTH**<br>**Strategy 5. (second opinion desired)** | | | | | | |
| % requiring 2nd opinion | 17.1 | 33.0 | 48.8 | 56.3 | 42.9 | 43.4 |
| % requiring 3rd opinion | 5.6 | 19.9 | 28.8 | 35.4 | 20.1 | 24.4 |
| **Strategy 6. (policy or desired)** | | | | | | |
| % requiring 2nd opinion | 21.0 | 40.7 | 64.6 | 78.9 | 74.2 | 62.4 |
| % requiring 3rd opinion | 6.4 | 24.2 | 37.2 | 47.6 | 27.5 | 32.1 |

© 2019 Piepkorn MW et al. *JAMA Network Open*

**eTable 3.** Four Second Opinion Strategies Based on Primary and Consulting Pathologists' Experience: Percentage Requiring Third Opinions

| **Strategy** | **Rate, %<br>M-Path Reference Consensus Diagnosis** | | | | | |
|---|---|---|---|---|---|---|
| | **Benign (I)** | **Moderately Dysplastic (II)** | **Severely Dysplastic/MIS (III)** | **pT1a melanoma (IV)** | **pT1b melanoma or greater (V)** | **Overall** |
| **Strategy 7. (1st, 2nd & 3rd general pathologists)** | | | | | | |
| % requiring 3rd opinion | 12.0 | 39.3 | 51.9 | 62.2 | 36.3 | 44.4 |
| **Strategy 8. (1 and 2nd general pathologists, 3rd dermatopathologists)** | | | | | | |
| % requiring 3rd opinion | 12.0 | 39.3 | 51.9 | 62.2 | 36.3 | 44.4 |
| **Strategy 9. (1st general pathologist, 2nd & 3rd dermatopathologists)** | | | | | | |
| % requiring 3rd opinion | 14.0 | 48.3 | 61.4 | 60.6 | 33.2 | 47.1 |
| **Strategy 10. (1st, 2nd & 3rd dermatopathologists)** | | | | | | |
| % requiring 3rd opinion | 15.2 | 51.7 | 52.3 | 48.5 | 27.8 | 41.2 |

© 2019 Piepkorn MW et al. *JAMA Network Open*
